# The buzz about bees and poverty alleviation: Identifying drivers and barriers of beekeeping in sub-Saharan Africa

**DOI:** 10.1371/journal.pone.0172820

**Published:** 2017-02-24

**Authors:** Deborah Ruth Amulen, Marijke D’Haese, Elizabeth Ahikiriza, Jacob Godfrey Agea, Frans J. Jacobs, Dirk C. de Graaf, Guy Smagghe, Paul Cross

**Affiliations:** 1 Department of Crop Protection, Ghent University, Ghent, Belgium; 2 Department of Livestock Industrial Resources, Makerere University, Kampala, Uganda; 3 Department of Agricultural Economics, Ghent University, Ghent, Belgium; 4 Department of Extension and Innovation Studies, Makerere University, Kampala, Uganda; 5 Laboratory of Molecular Entomology and Bee Pathology, Ghent University, Ghent, Belgium; 6 School of Environment, Natural Resources and Geography, Bangor University, Gwynedd, United Kingdom; University of Cologne, GERMANY

## Abstract

The potential of beekeeping to mitigate the exposure of rural sub-Sahara African farmers to economic stochasticity has been widely promoted by an array of development agencies. Robust outcome indicators of the success of beekeeping to improve household well-being are unfortunately lacking. This study aimed to identify the key drivers and barriers of beekeeping adoption at the household level, and quantified the associated income contribution in three agro-ecological zones in Uganda. Beekeepers were generally the most economically disadvantaged people in the study areas and tended to adopt beekeeping following contact with non-government organisations and access to training. Whilst incomes were not statistically lower than their non-beekeeping counterparts; their mean household well-being scores were significantly lower than non-beekeeping households. The inability of beekeeping to significantly improve well-being status can in part be attributed to a lack of both training in bee husbandry and protective equipment provision such as suits, gloves and smokers. These are critical tools for beekeepers as they provide the necessary confidence to manage honey bees. Rather than focussing solely on the socio-economic conditions of farmers to effectively adopt beekeeping, future research should also attempt to evaluate the effectiveness of development agencies’ provision to the beekeeping sector.

## Introduction

The impact of environmental and economic shocks on the rural poor, who depend upon their own local food system to survive, can have profound implications for their livelihood and welfare security [1]. A diversified livelihoods portfolio is considered by many as a more resilient system to manage risk [2,3]. A household’s ability to diversify into more resilient income streams is contingent upon ownership, control and access to key livelihood assets such as finance, markets and education [4]. As a means of improving rural incomes, beekeeping has been promoted among many developing countries as a diversified livelihood option by international organisations, governments and NGOs [5,6].

Beekeeping offers multiple potential benefits to the rural poor such as increased household income streams [7,8], nutritional and medicinal products for sale or home use [9] as well as improving pollination services essential for increased crop yields [10,11]. Whilst beekeeping appears to have a contribution to make to rural livelihoods, its purported production potential in sub-Saharan Africa remains relatively untapped. For example, Kenya’s production potential is estimated to be 100,000 tonnes of honey per year, but only 14.6% of this is realized [12–14]. Similarly, Uganda harvests only 1% of its estimated production potential of 500,000 tonnes of honey per year [15]. Furthermore, Africa’s share of the world honey trade also remains low [16]. However, it is nonetheless thought to hold a competitive advantage in the organic and fair trade sectors [17], suggesting that substantial opportunities exist for rural households to improve their economic resilience.

Uganda is among the five countries in sub-Saharan Africa licensed to export honeybee products to the European Union [18]. In spite of this opportunity to develop the export market, Uganda has failed to meet both its export quota to the EU as well as home grown demand for honey [18], due to low domestic productivity and weak beekeeping adoption rates [15].

Adoption of new agricultural technologies in sub-Saharan Africa is typically contingent upon access to appropriate technical information and provision of reliable costs and benefits associated with the activity [19,20]. Several studies suggest beekeeping adoption is contingent upon multiple factors all of which may interact: on-farm income; level of savings and access to credit; cash generation; household food and medicine provision; availability of agricultural extension services and membership of farmers’ groups [12,15,21,22].

This study sought to identify the key drivers of beekeeping adoption, and aimed to quantify the degree to which beekeepers’ household well-being differed from non-beekeepers. The study objectives were thus to: 1) quantify beekeeper household status using a well-being index, 2) identify key factors drivers and barriers of beekeeping adoption, and 3) quantify the relative contribution of beekeeping to household income.

## Materials and methods

### Study area and data collection

The study was carried out in the primary honey producing areas of Uganda [23] which included the West Nile (Arua district), the Eastern (Soroti district) and the mid-Northern (Kitgum district) agro-ecological zones as clustered by [24,25]. Before commencing the study, ethical approval was obtained from the ethics review committee of the College of Veterinary Medicine, Animal Resources and Biosecurity, Makerere University (No. SBLS.ADR.2016). Consent forms were signed prior to each respondent being interviewed and they were advised that they were free to participate or withdraw at any point during the interview.

### Sampling procedure

Multi-stage sampling, using purposive and random-stratified techniques, was used to identify beekeeping and non-beekeeping households. The three agro-ecological zones were purposively selected based on mean annual honey yields. The West Nile-Arua district (84,320 kg) was classified as a relatively high producer, the mid-Northern–Kitgum district (27,500 kg) as moderate, and the Eastern-Soroti district (<16,310 kg) as a low producer [26]. Beekeeper respondents were randomly selected from a list obtained from the Uganda National Apiculture Development Organization (TUNADO), and stratified by agro-ecological zone. Non-beekeepers were randomly selected from a list of farmers provided by each district agricultural office.

The beekeepers’ list comprised 630 beekeeping households of which 166 were selected for interview (Table 1). A semi-structured questionnaire was administered to any adult beekeeper and non-beekeeper in each household.

**Table 1:** Summary of sampled households with number of beekeepers and non-beekeepers in the three agro-ecological zones in Uganda for this study.

Agro-ecological zone	Beekeepers	Non-beekeepers	Total
mid-Northern—Kitgum	38	30	68
Eastern—Soroti	66	51	117
West Nile—Arua	59	57	116
Total	163	138	301

A set of 34 variables was analysed to identify any significant factors influencing choice of farm enterprise, beekeeping adoption and attitudes towards beekeeping (S1 Table). Descriptive and inferential statistical tools such as the Pearson chi-square likelihood ratios and Levene’s test of equal variances were used to show which t-statistic to consider during comparison of beekeepers with non-beekeepers. Household well-being variables (S2 Table) selected from a list adapted from [27] were validated during focus group discussions and the subsequent data was aggregated to generate a household well-being index using a categorical principal component analysis (CATPCA) [28]. Factor scores were generated using spline ordinal transformation and dimension one was used to calculate the household well-being index, whereby a value of 3 indicated a relatively wealthy attribute score and 1 indicated the least wealthy score. The above analyses were performed in Statistical Package for the Social Sciences (SPSS) version 22 [29].

To explore predictors of beekeeping adoption and intensity of beekeeping, a two-stage Heckman model was applied [30]. It was chosen for its ability to correct sample selection biases [31,32]. Our study tried to avoid biases resulting from correlation of error terms and simultaneously omitted variables. The first model predicted the probability of a farmer adopting beekeeping using a probit maximum likelihood function for both beekeepers and non-beekeepers. The second model used an ordinary least squares estimation equation for the intensity of beekeeping (number of beehives owned) as determined by the farm household asset endowments and characteristics with the inverse Mill’s ratio term as an added variable to reveal the probability of an observation belonging to the selected sample group. A significant Mill’s lambda ratio indicates the presence of sample selection biases and that they were corrected [31,32]. The above analyses were performed in Stata 13 statistical software [32]. Furthermore, beekeepers were classified into small scale and large scale producers based on mean number of beehives owned. Those beekeepers who had beehives less than the mean were classified as small scale while large scale beekeepers were above the mean.

## Results

### Socio demographic characteristics of respondents

There was no significant difference in age and household size between beekeepers and non-beekeepers (t = 1.02) (Table 2). Beekeepers’ mean annual income was significantly lower than non-beekeepers (t<0.05). The proportion of farmers owning land and the average farm size were not significantly different between beekeeping and non-beekeeping households (t = -0.14). The reported mean land acreage per household (9.22 for beekeepers and 9.44 for non-beekeepers) was higher in the study area compared to the national average of 2.8 acres per household [33]. Few beekeepers (4.2%) had attained secondary or tertiary education compared to 48.5% of non-beekeepers (Table 2).

**Table 2:** Socio-demographic characteristics of households as measured with continuous and categorical variables.

Characteristics	Beekeeper (mean±s.e.)	Non-beekeeper (mean±s.e.)	t-statistic
**Age**	44.62±1.21	42.84±1.24	1.02
**Number of household members**	10.37±0.38	10.30±0.39	0.13
**Number of land acres**	9.22±1.29	9.44±0.88	-0.14
**Number of cattle**	4.72±0.46	4.68±0.46	0.06
**Number of sheep**	1.59±0.37	0.55±0.16	2.42**
**Number of goats**	4.97±0.41	3.93±0.40	1.80*
**Number of pigs**	1.17±0.26	0.36±0.10	2.67***
**Number of poultry**	23.82±2.43	12.33±0.86	4.17***
**Land allocated to crops**	5.48±0.43	5.21±0.37	0.45
**Land allocated to livestock**	2.34±0.34	1.53±0.14	1.96**
**Annual crop income**	382.97±72.67	245.07±19.54	1.70**
**Annual livestock income**	89.36±9.90	325.42±50.41	-4.96***
**Annual non-farm income**	90.45±16.42	320.50±62.50	-3.82***
**Total household income**	605.82±74.19	870.47±81.82	-2.40**
	Beekeeper (%, n = 163)	Non-beekeepers (%, n = 138)	Chi-Square value df = 294
**Gender (comparison of gender distribution between beekeepers yes = 1 & non-beekeeper no = 0)**			9.373***
**Females**	21.7	37.7	
**Males**	78.3	62.3	
**Education (comparison of education level between beekeeper yes = 1 & non-beekeepers no = 0)**			90.479***
**No formal education**	59.6	19.6	
**Primary education**	36.1	31.9	
**Secondary education**	3.6	39.1	
**Tertiary education**	0.6	9.4	
**Main income sources (comparison of main income sources between beekeeper yes = 1 & non-beekeepers no = 0)**			9.604***
**On-farm income sources**	85.5	71.0	
**Off-farm income**	7.2	15.2	
**Non-farm income**	7.2	13.8	
**Land ownership (comparison of land ownership between beekeeper yes = 1 & non-beekeepers no = 0)**			1.926
**Landownership**	83.7	88.4	

*** denotes the mean or percentage difference between beekeepers and non-beekeepers is significant at 1%,

** the mean or percentage difference between beekeepers and non-beekeepers is significant at 5%,

* the mean or percentage difference between beekeepers and non-beekeepers is significant at 10%

**Fig 1:**
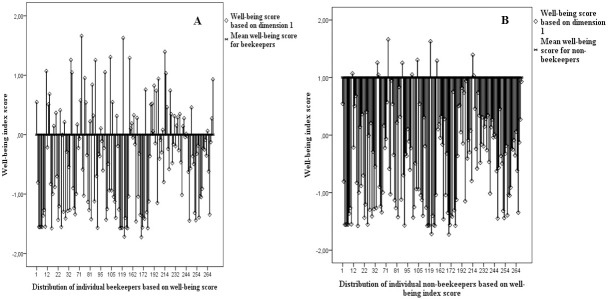
Comparing the mean well-being score for beekeepers and non-beekeepers A) indicates a low mean score which according to the study measurements signify less wealthy households, while B) shows a higher mean score implying non-beekeeping households were slightly wealthier than the beekeepers. All measurements based on dimension one scores.

Eleven of the 16 well-being indicators explained variation in household well-being across the three agro-ecological zones (Table 3). These were as follows in order of descending proportional variance: *1) ownership of new clothes and shoes*, *2) food shortage*, *3) ability of a household to send children to school*, *4) household member hired as casual labourer*, *5) number of meals per day*, *6) type of house owned*, *7) sleeping on a mattress*, *8) ownership of animals*, *9) household’s ability to hire labour*, *10) member of household in off-farm employment and 11) use of rare items like sugar and cooking oil*. The mean well-being index (calculated based on the CATPCA) of beekeeping households was significantly lower than that of non-beekeepers, suggesting that beekeeping households were relatively less wealthy compared to non-beekeepers (Fig 1) (S3 Table). For instance, a majority of beekeeping households (53%) slept hungry compared to 12% among non-beekeepers. A high number of beekeeping households (42%) also reported having faced a food shortage in their households that lasted more than two months compared to 17% of the non-beekeeping households. Non-beekeepers had a higher proportion of cattle ownership compared to beekeepers, and a majority (54%) of beekeepers had no off-farm employment compared to 12% of the non-beekeepers. Other well-being indicators revealed that provision of everyday casual labour was high among beekeepers. A majority of beekeepers (88%) owned grass-thatched houses compared to 37% among non-beekeepers, with 72% of the beekeepers sleeping on polythene or mats as opposed to mattresses compared to only 23% of non-beekeepers.

**Table 3:** Factor loadings for well-being indicators based on varimax rotation.

Variable	Centroid Coordinates		Mean variance
	Dimension 1	Dimension 2	
**Ownership of new clothes and shoes**	0.71	0.02	0.37
**Experienced food shortage and how long it lasted**	0.69	0.01	0.35
**Children in the household in school**	0.68	0.01	0.34
**Any member of household hired as casual labourer**	0.63	0.01	0.32
**Meals per day**	0.59	0.01	0.30
**Type of house owned**	0.57	0.00	0.28
**Sleep on mattress**	0.53	0.02	0.27
**Which animals were owned**	0.50	0.03	0.27
**Household hires labour**	0.49	0.01	0.25
**Any member of household in off-farm employment**	0.48	0.02	0.25
**Use of rare items like sugar, cooking oil**	0.44	0.01	0.22
**Marital status of household head**	0.08	0.05	0.07
**Ownership of any scarce assets**	0.00	0.62	0.31
**Age**	0.01	0.57	0.29
**Land ownership of the household**	0.01	0.11	0.06
**Membership in any groups**	0.01	0.07	0.04
**Active total**	6.40	1.56	3.98
**% of variance**	40.00	9.74	24.87

In this model the Cronbach’s Alpha for dimension 1 is 0.90, dimension 2 is 0.337, and the total is 0.931.

Most beekeepers (93%) were affiliated to a farmer group compared to non-beekeepers (65%). Significantly fewer beekeepers (48%) were members of savings groups compared to non-beekeepers (67%). Beekeepers were more likely to have adopted beekeeping (97%) if access to beekeeping extension services (access to relevant information) was available compared to the non-beekeepers’ likelihood (70%) to start other on-farm enterprises. Neither access to routine extension officer visits nor market information varied significantly between beekeepers and non-beekeepers (Table 4). Beekeeping extension services were mainly provided by NGOs and government agricultural extension departments, whilst agricultural extension services for other farm enterprises to non-beekeepers were mainly provided by government agricultural extension services and private consultants (Table 4).

**Table 4:** Accessibility of agricultural extension services.

Type of agricultural extension services	Non-beekeepers n = 138 (%, yes = 1)	Beekeepers n = 163 (%, yes = 1)	Chi-square value (df = 1)
Agricultural extension services	69.57	96.99	43.36***
Training on agricultural enterprise & beekeeping management	31.1	87.95	103.30***
Training on agricultural & beekeeping product processing	37.68	58.43	12.99***
Routine extension agent visits	36.96	46.39	2.75
Agricultural input & beekeeping equipment support	46.38	87.35	58.88***
Agricultural and beekeeping products market information	63.04	59.04	0.51
Sources of agricultural extension services			
NGOs	1.45	55.06	132.048***
Government	74.64	51.81	16.701***
Private consultation and community based services	59.42	14.45	67.079***
Fellow farmers	51.45	28.92	16.062***
Media	18.84	6.63	10.516***

*** The mean difference between beekeepers and non-beekeepers is significant at 1%,

### Drivers and barriers to beekeeping adoption

The farmers’ decision to diversify their on-farm enterprises was mainly driven by the need to fulfil their household nutritional needs (yes = 1, no = 0, n = 280/296, 92%) and income (yes = 1, no = 0, n = 269/296, 91%). Other drivers of on-farm enterprise diversification included availability of market for products (yes = 1, no = 0, n = 210/296, 71%), on-farm labour demands (yes = 1, no = 0, n = 91/296, 31%) and presence of local knowledge and traditional uses for the particular on-farm enterprise (yes = 1, no = 0, n = 60/296, 20%). The farmers’ diversification into beekeeping was mainly driven by the perceived higher income earning potential from hive products (59%), after seeing other farmers keeping bees (51%), as well as information and support received from government departments (50%) and non-government organizations.

The non-beekeepers were disinclined to adopt beekeeping due to limited beekeeping knowledge (62%), fear of defensive honey bees (59%), insufficient capital to purchase inputs (31%) and limited land availability (24%). Several non-beekeepers were unsure whether beekeeping could be profitable (16%) and cited poor market access for hive products (15%) as deterrents to beekeeping adoption. Interestingly, in response to a Likert scale set of questions, the majority of non-beekeeping respondents was either ‘very interested’, ‘interested’ or ‘somewhat interested’ in adopting beekeeping, suggesting an untapped source of new beekeepers (n = 92/130, 71%).

### Predictors of beekeeping technology adoption

Probit modelling allowed the identification of significant associations between farmers’ socio-economic characteristics and likelihood to adopt beekeeping (Table 5). Several probit models with different specifications were estimated in order to test the robustness and significance of the coefficients. Male farmers and those who had none or only primary education were more likely to be beekeepers. Households with comparatively lower well-being index scores were more likely to be beekeepers. Membership to a farmer’s group increased a farmer’s likelihood of becoming a beekeeper. Contact with NGOs also significantly increased the likelihood to adopt beekeeping (Table 5).

**Table 5:** Step 1: Probit estimation of socio-economic predictors of beekeeping adoption.

Explanatory variable	Model 1 Coefficients (s.e.)	Model 2 Coefficients (s.e.)	Model 3 Coefficients (s.e.)
Age	-0.004 (0.008)	<0.001 (0.007)	-0.005 (0.008)
Gender (1: male, 0: female)	0.840 (0.258) ***	0.555 (0.248) **	0.730 (0.272) **
Primary education (1: yes)	-0.775 (0.239) ***		0.344 (0.270)
Secondary and tertiary education (1: yes)	-1.903 (0.346) ***		-1.149 (0.368) ***
Household size (number)	0.012 (0.024)	0.023 (0.023)	0.008 (0.023)
Land acreage (acres)	0.004 (0.008)	0.008 (0.007)	0.008 (0.008)
Annual income (Ugandan shillings)	-3.640 (4.710)		-5.970 (6.130)
Household well-being index (score)		-0.702 (0.116) ***	-0.540 (0.158) ***
Membership to farmer group (1: yes)	0.734 (0.294) **	1.062 (0.291) ***	0.919 (0.305) **
Contact with NGO (1: yes)	2.454 (0.341) ***	2.565 (0.338) ***	2.379 (0.341) ***
Constant	-0.931 (0.487)	-2.055 (0.498)	-1.779 (0.533) ***

Model 1: Wald chi2 (8) = 40.64 prob (chi2) <0.001, Model 2: Wald chi2 (9) = 28.62 prob (chi2) <0.001, Model 3: Wald chi2 (8) = 28.93 prob (chi2) <0.001

Note:

** at 5%, and

*** at 1%. Number of observations = 301, censored = 138, uncensored = 163.

### The intensity of beekeeping adoption

The mean number of hives per beekeeper was 22.87 (s.d. = 22.73; min 2; max 192). A substantial proportion of beekeepers had only one to three years beekeeping experience (43.4%, n = 72/166), followed by 31.3% (n = 52/166) with four to seven years, and only 25.3% (n = 42/166) with eight years or more. Using three ordinary least square models, the robustness and significance of coefficients measuring intensity of beekeeping adoption was estimated. The intensity of beekeeping adoption (i.e. the number of hives owned) was primarily dependent upon the beekeeper’s years of experience and membership to a savings or credit group (Table 6).

**Table 6:** Step 2: Estimation of beekeeping adoption intensity.

Explanatory variable	Model 1 Coefficients (s.e.)	Model 2 Coefficients (s.e.)	Model 3 Coefficients (s.e.)
Years of experience in beekeeping	4.974 (1.656) ***	4.966 (1.677) ***	4.897 (1.166) ***
Land acreage (acres)	-0.095 (0.105)	-0.092 (0.106)	-0.093 (0.105)
Dummy farmer interested in beekeeping (1: yes)		8.182 (12.063)	8.034 (12.061)
Membership to farmer group (1: yes)	4.441 (6.381)	5.077 (6.236)	4.772 (6.311)
Membership to savings and credit group (1: yes)	6.906 (3.462) **	6.608 (3.463) **	6.670 (3.462) **
Access to beehives donations through NGO and government (1: yes)	0.210 (5.074)	0.616 (5.014)	0.443 (5.043)
Distance to market (kilometres)	0.5197 (0.234) *	0.495 (0.236) *	0.498 (0.236)
Dummy West Nile ecological zone	8.677 (4.851)	8.663 (4.846)	8.852 (4.869)
Dummy Eastern ecological zone	-2.524 (4.830)	-2.907 (4.799)	-2.765 (4.821)
Mills Lambda ratio	-1.742 (4.130)	-1.043 (4.084)	-1.754 (4.379)
Constant	0.930 (0.487)	-15.584 (15.704)	17.228 (10.318)
rho	-0.086	-0.052	-0.087
Sigma	20.237	20.203	20.209

Model 1: Wald chi2 (8) = 26.47 prob (chi2) <0.001, Model 2: Wald chi2 (9) = 40.55 prob (chi2) <0.001, Model 3: Wald chi2 (9) = 41.23 prob (chi2) <0.001

Note:

* refers to a significance at 10%,

** at 5%, and

*** at 1%. Number of observations = 301, censored = 138, uncensored = 163.

Beekeepers acquired most of their top bar and frame hives (KTB and Langstroth, respectively) through donations. Traditional beehives tended to be locally made by the beekeepers (Table 7).

**Table 7:** Provision of beehives.

Type of beehive	Number of beekeepers	Proportion bought (%)	Proportion donated (%)	Proportion locally made (%)	Proportion co-funded (%)
*Traditional beehives*					
Log hives	150	25	4	70	1
Pot hives	35	41	19	0	40
*Top bar hives*					
Kenya top bar (KTB)	109	10	84	2	4
*Frame hives*					
Langstroth	34	12	88	0	0

Honey was the main product harvested, followed by beeswax and propolis. Beekeeping contributed about 7% to annual household incomes (Table 8). So, this may indicate that if beekeeper households did not have beekeeping to supplement their income, they would be 7% financially worse-off than they are currently.

**Table 8:** Products harvested, current income benefits and unit product prices from beekeeping.

Variable	Annual yield per beekeeper Mean ± s.e. (n = 163)
Products (annual yields, kg)	
Honey	13.42±1.39
Beeswax	3.51±1.26
Propolis	0.19±0.80
Current income benefit (annual income, USD)	
Total household income	615.48±74.18
Honey	32.10±3.43
Beeswax	10.33±4.50
Propolis	0.58±0.34
Total beekeeping income	43.01±6.92
Proportion of beekeeping income	0.69
Unit prices of product (USD/kg)	
Honey	2.61±0.14
Beeswax	3.01±0.36
Propolis	4.00±1.19

## Discussion

For the first time to the authors’ knowledge, this study has attempted to categorise and quantify the impact of beekeeping on household well-being. However, disaggregating the influence of beekeeping on well-being without knowledge of the pre-existing socio-economic conditions of the household renders causality difficult. Nonetheless, this study has identified key drivers and barriers of beekeeping adoption and their relative impact on household well-being.

Before further discussing the implications of this study, it is worthwhile considering the following caveat. Whilst the use of all livelihood asset capitals in this study facilitated the contextualization of beekeeping adoption drivers, other important factors that may have influenced farmers’ livelihood choices such as the influence of institutional, economic, social and political processes were not included. Such processes and their interactions are complex and consequently beyond the scope of this current study.

In this study, all beekeepers were farmers but not all farmers were beekeepers and yet beekeepers were significantly less educated and had a well-being index score lower than their non-beekeeping counterparts. Low educational attainment in sub-Saharan Africa has previously been used as a proxy measure of poverty [34] and there are obvious correlates between education and the well-being outcome in our study. Given that beekeeping farmers were comparatively more disadvantaged in terms of their overall well-being, it is somewhat surprising that they went to the additional cost of acquiring hives in the first place. It is widely agreed in the agricultural technology adoption literature that farmers who are relatively wealthier than their counterparts tend to more readily accept the associated risks of new technology adoption [4].

One possible partial explanation of such risk-taking resides in the development agencies’ programme prioritisation of key recipients, who identify and target the economically most disadvantaged in society as a means of maximising programme impact on poverty alleviation [35–37]. Consequently, most of the top bar and frame hives owned by respondent beekeepers were acquired through either NGO or Ugandan Government donations. Whilst contact with development organisations was associated with hive type ownership and was a key factor in farmer adoption of beekeeping, the hive management skills of beekeepers were independent of any previous contact with NGOs or Government departments, suggesting that skills remained undeveloped.

Sufficient access to knowledge transfer outlets such as extension service officers and/or NGO programmes was critical in the adoption and continuation of beekeeping. Many extension services have been sub-contracted by the Ugandan Government to private service providers as a means to offset the staffing costs of frontline agricultural extension provision, particularly in remote areas such as Northern Uganda [38]. Possibly as a consequence of cost-saving initiatives, the provision of adequate training in beekeeping was generally absent. The importance of training in driving technology adoption in this study is commensurate with the findings of several other studies [20,39–41]. Farmer group membership was found to be an important factor in beekeeping adoption. This is probably due to the demands frequently made by extension service providers (NGOs and Government extension departments), who tend to prefer training and equipment provision to be directed towards farmer groups rather than individual farmers in order to maximise economies of scale [20,42].

Factors that inhibited beekeeping adoption rates included insufficient knowledge, fear of defensive bee behaviour and limited financial capital. The role of knowledge acquisition on technology adoption rates, especially as a free-flowing exchange of information within and between communities, has been previously identified [43]. Appropriate and repeated training of beekeepers in honeybee behaviour management would help to address this challenge [44].

Whilst beekeeping adoption has generally been understood to entail the presence of at least one hive on the farm, this is not necessarily a sufficiently robust indicator of successful adoption. For instance, the adoption intensity (the relative number of hives per beekeeper) is also critical in determining the impact of a government or NGO-driven beekeeping programme. The majority of beekeepers in this study, as in previous studies [12–14], were small-scale beekeepers (<22 hives) primarily using traditional hives and generating low honey yields. In this study we found that the income contribution of beekeeping to households was not significantly high. Girma & Gardebroek [42] found the beekeeper income contribution to rural households to be high in Ethiopia, whilst Leisher et al [45] found it to be lower, suggesting that income contribution of beekeeping to rural livelihoods varies regionally and beekeeping alone may not be sufficient to alleviate relative impoverishment (as defined by the above authors) among the rural poor in Uganda.

A key component of the success or failure of the beekeeping adoption rate in this study was the lack of relevant training regarding effective hive management. Whilst the donation of beehives from NGOs increased the likelihood of farmers to adopt beekeeping, the ultimate success and continuation of beekeeping was contingent upon access to appropriate training, frequency of delivery and provision of protective equipment. Most beekeepers had been given only one day’s training, and this was delivered in a classroom context without any practical exposure. The quality and quantity of such training were not quantified in this study, but many beekeepers reported that they were unable to effectively use the top bar hive and preferred to use the fixed-comb style hive. This has important implications for NGOs who continue to supply hives throughout sub-Saharan Africa, as it would appear that the donation of hives to the exclusion of protective equipment and training is likely to fail to improve beekeeping households’ well-being. Furthermore, a lack of appropriate training and supply of protective equipment may lead to the undermining of farmer confidence in both the honey product sector and NGOs as a positive vector of livelihood change.

## Concluding remarks

Whilst ‘beekeeping as a poverty alleviating activity’ has been widely promoted by international development agencies, national and local governments and an array of NGOs as a panacea to poverty alleviation, this study suggests that beekeeping has not significantly improved the farmers’ well-being. Critical requirements of successful beekeeping adoption by farmers (as identified by the farmers) are bee husbandry knowledge and protective equipment. However, what they are typically provided with, is an inexhaustible supply of modern hives and insufficient training in hive management. Rather than focussing solely on the plight of farmers to effectively adopt beekeeping, future research should attempt to evaluate the effectiveness of development agencies’ provision to the beekeeping sector.

## Supporting information

S1 Table Determinant factors of beekeeping adoption. (DOCX)

S2 Table Household well-being score card used. (DOCX)

S3 Table Summary statistics of household wellbeing indicators. (DOCX)
