# Supplementary material for: The buzz about bees and poverty alleviation: Identifying drivers and barriers of beekeeping in sub-Saharan Africa
Source: PLoS One. 2017 Feb 24;12(2):e0172820. doi: 10.1371/journal.pone.0172820 (PMC5325527; doi:10.1371/journal.pone.0172820)
Supplement: S1 Table — (DOCX) [file pone.0172820.s001.docx]

| Table 1: Determinant factors of beekeeping adoption | | |
| --- | --- | --- |
|  | Variables (Factors) | Responses |
|  | *Household characteristics* |  |
| 1 | Age | numeric |
| 2 | Number of household members | numeric |
| 3 | Household income | numeric |
| 4 | Well-being index | numeric |
| 5 | Land acreage | numeric |
| 6 | Gender | (1=male; 0=females) |
| 7 | Education | (no formal education 1=yes, 0=no; primary 1=yes, 0=no; secondary 1=yes, 0=no; tertiary 1=yes, 0=no; secondary & tertiary 1=yes, 0=no) |
| 8 | Main income sources | (1=on-farm, 2=off farm, 3=non-farm) |
| 9 | Land ownership | (1=own land, 2=do not own, 3=share land) |
|  | *Access to agricultural & beekeeping extension* |  |
| 10 | Agricultural extension services & beekeeping | (1=yes; 0=no) |
| 11 | Training on management of agricultural enterprises & beekeeping | (1=yes; 0=no) |
| 12 | Training on agricultural & beekeeping products processing | (1=yes; 0=no) |
| 13 | Routine extension agent visits | (1=yes; 0=no) |
| 14 | Agricultural inputs & beekeeping equipment support | (1=yes; 0=no) |
| 15 | Agricultural and beekeeping products market information | (1=yes; 0=no) |
|  | *Sources of extension services* |  |
| 16 | NGOs | (1=yes; 0=no) |
| 17 | Government | (1=yes; 0=no) |
| 18 | Private consultation and community based | (1=yes; 0=no) |
| 19 | Fellow farmers | (1=yes; 0=no) |
| 20 | Media | (1=yes; 0=no) |
|  | *Drivers to farmer diversification* |  |
| 21 | Parents | (1=yes; 0=no) |
| 22 | Access to training | (1=yes; 0=no) |
| 23 | Personal interest | (1=yes; 0=no) |
| 24 | Prospects of high income | (1=yes; 0=no) |
| 25 | NGO and government | (1=yes; 0=no) |
| 26 | Seeing fellow farmers start beekeeping | (1=yes; 0=no) |
|  | *Barriers to farmer diversification* |  |
| 27 | Limited knowledge | (1=yes; 0=no) |
| 28 | Fear of aggressiveness | (1=yes; 0=no) |
| 29 | Limited capital | (1=yes; 0=no) |
| 30 | Limited land space | (1=yes; 0=no) |
| 31 | No interest in beekeeping | (1=yes; 0=no) |
| 32 | Doubt profitability of beekeeping | (1=yes; 0=no) |
| 33 | No market | (1=yes; 0=no) |
| 34 | Non-beekeeper’s attitudes towards beekeeping | (1=not at all interested; 2=not interested; 3=somewhat interested; 4=Interested; 5=very interested) |
