# Supplementary material for: The buzz about bees and poverty alleviation: Identifying drivers and barriers of beekeeping in sub-Saharan Africa
Source: PLoS One. 2017 Feb 24;12(2):e0172820. doi: 10.1371/journal.pone.0172820 (PMC5325527; doi:10.1371/journal.pone.0172820)
Supplement: S2 Table — (DOCX) [file pone.0172820.s002.docx]

| Table 2: Household well-being score card used | | | |
| --- | --- | --- | --- |
|  | Indicator | Category | Description |
| 1 | Food | 3 | Two meals a day |
|  |  | 2 | One meal per day |
|  |  | 1 | Sleep hungry at times, beg for food, can't afford food |
| 2 | Food security | 3 | Have not experienced a period of food shortage within the last year |
|  |  | 2 | Have experienced a period of food shortage within the last year, lasted <2months |
|  |  | 1 | Have experienced a period of food shortage within the last year, lasted >2months |
| 3 | Feed | 3 | Frequently buys sugar, meat and fries food |
|  |  | 2 | Occasionally buys sugar, meat and fries food |
|  |  | 1 | Rarely buys sugar, meat and fries food |
| 4 | Animals | 3 | Somebody in the household has cattle |
|  |  | 2 | Nobody in the household has cattle, but they have goats, sheep, pigs, poultry) |
|  |  | 1 | Nobody in the household has any animals |
| 5 | Land | 3 | Own land (leasehold, customary and freehold) more than 5 acres (2.0ha) |
|  |  | 2 | Own Land (leasehold, customary and freehold) less than 5 acres (2.0ha) |
|  |  | 1 | Do not own land or own less than 1acre (0.4ha) |
| 6 | Off-farm employment | 3 | Somebody in the household has 'high entry costs' a job like professional, business (trading, transport) (more paying) |
|  |  | 2 | Somebody from the household has off-farm income like tailoring, building, craft making, brewing beer, charcoal, selling food |
|  |  | 1 | Nobody from the household is engaged in off-farm employment |
| 7 | Labour | 3 | Nobody from the household works for others as a casual labourer |
|  |  | 2 | Somebody from the household works for others as casual labourer, but either only 3 months or less per year or more than three months but not more than once a week |
|  |  | 1 | Somebody from the household works for others as a casual labourer more than 3 months per year or less than 3 months per year but almost everyday |
| 8 | Hire labour | 3 | Hire labourers for at least two of the following tasks: land clearing, ploughing, planting, wedding, harvesting |
|  |  | 2 | Sometimes hire labour |
|  |  | 1 | Do not hire labourers or hire labourers for one task only |
| 9 | Housing | 3 | Have houses with bricks or plastered walls and iron or tile roof |
|  |  | 2 | Have houses with unburned bricks old iron sheets, grass thatched but very neat |
|  |  | 1 | Have houses with Mud walls, grass thatched roofs, need repairs |
| 10 | Education | 3 | Have children or somebody in a private school |
|  |  | 2 | Have not had anyone in private school or secondary school |
|  |  | 1 | Children are not in school |
| 11 | Dressing | 3 | Own shoes, new clothes within the last 3 months |
|  |  | 2 | Does not own shoes or at least got new clothes 6 months ago, children are almost naked |
|  |  | 1 | Both woman and children last had new clothes a year ago |
| 12 | Bedding | 3 | Sleep in a bed and mattress, even the children |
|  |  | 2 | Only parents sleep on a mattress, children on a mat or polythene |
|  |  | 1 | All of them on a polythene, or mat no one owns a mattress |
| 13 | Marital status | 3 | Household head is male or married woman |
|  |  | 2 | Household head is a widow, single or divorced woman |
|  |  | 1 | Household head is orphaned child, youth |
| 14 | Age | 3 | Old (56-91 years) |
|  |  | 2 | Middle-aged (36-55 years) |
|  |  | 1 | Youth (17 -35 years) |
| 15 | Social capital | 3 | In many associations (farmer groups, burial groups, community groups) |
|  |  | 2 | Not in any association (farmer groups, community groups) |
|  |  | 1 | Unable to join a burial group (cannot pay subscription) |
| 16 | Scarce assets | 3 | Owns a motorcycle, bicycle, radio, mobile phone |
|  |  | 2 | Owns at least small radio, and a bicycle, mobile phone |
|  |  | 1 | Do not own any of the above |
|  | Table modified from (27) | | |
