# Supplementary material for: The buzz about bees and poverty alleviation: Identifying drivers and barriers of beekeeping in sub-Saharan Africa
Source: PLoS One. 2017 Feb 24;12(2):e0172820. doi: 10.1371/journal.pone.0172820 (PMC5325527; doi:10.1371/journal.pone.0172820)
Supplement: S3 Table — (DOCX) [file pone.0172820.s003.docx]

|  |  |  |  |  |  |
| --- | --- | --- | --- | --- | --- |
| Table 3: Summary statistics of household wellbeing indicators | | | | | |
|  | Variables | All farmers, n=304, Percentage | Non-beekeepers, n=138,  Percentage | Beekeepers, n=166, Percentage | Chi-square value df =2 |
| 1 | Meals per day vs beekeeper | | |  | 59.175*** |
|  | sleep hungry | 34.54 | 12.32 | 53.01 |  |
|  | one meal per day | 37.83 | 45.65 | 31.33 |  |
|  | two meals per day | 27.63 | 42.03 | 15.66 |  |
| 2 | Experienced food shortage and how long it lasted vs beekeeper | | | | 34.31*** |
|  | food shortage >2 months | 30.26 | 16.67 | 41.57 |  |
|  | food shortage <2 months | 34.87 | 32.61 | 36.75 |  |
|  | no food shortage within last year | 34.87 | 50.72 | 21.69 |  |
| 3 | Type of animals owned vs beekeeper | | | | 7.014** |
|  | no animals in household | 25.33 | 24.64 | 25.90 |  |
|  | no cattle but goats, sheep and pigs | 29.93 | 23.19 | 35.54 |  |
|  | owns cattle | 44.74 | 52.17 | 38.55 |  |
| 4 | Land ownership of the household vs beekeeper | | |  | 1.455 |
|  | do not own and share | 14.47 | 12.32 | 16.27 |  |
|  | own <5 acres | 30.92 | 29.71 | 31.93 |  |
|  | own >5 acres | 54.61 | 57.97 | 51.81 |  |
| 5 | Any member of household in off farm employment vs beekeepers | | | | 74.804*** |
|  | no off farm employment | 35.20 | 12.32 | 54.22 |  |
|  | small paying business | 22.04 | 52.90 | 40.36 |  |
|  | someone in high paying business | 2.96 | 34.78 | 5.42 |  |
| 6 | Any member of household hired as casual labourer vs beekeeper | | | | 7.379** |
|  | casual labourer everyday | 26.32 | 21.01 | 30.72 |  |
|  | casual labourer <once a week | 33.22 | 30.43 | 35.54 |  |
|  | nobody as casual labourer | 40.46 | 48.55 | 33.73 |  |
| 7 | The household hires labour vs beekeeper | | |  | 19.471*** |
|  | do not hire labour | 32.57 | 20.29 | 42.77 |  |
|  | sometimes hire labour | 41.12 | 44.93 | 37.95 |  |
|  | hire labour | 26.32 | 34.78 | 19.28 |  |
| 8 | Type of house owned vs beekeeper | |  |  | 88.471*** |
|  | grass thatched roof, mud walls | 64.80 | 36.96 | 87.95 |  |
|  | unburnt bricks old iron sheets, grass neat | 23.03 | 38.41 | 10.24 |  |
|  | brick houses and iron roof | 12.17 | 24.64 | 1.81 |  |
| 9 | Children in the household school vs beekeeper | | |  | 15.197*** |
|  | no children in school | 31.25 | 21.74 | 39.16 |  |
|  | no child in private school or secondary | 32.89 | 31.88 | 33.73 |  |
|  | children in private school | 35.86 | 46.38 | 27.11 |  |
| 10 | Ownership of new clothes and shoes vs beekeeper | | | | 58.898*** |
|  | no new clothes >1 year | 44.08 | 22.46 | 62.05 |  |
|  | no new clothes <6 months’ children almost naked | 33.22 | 38.41 | 28.92 |  |
|  | own shoes, new clothes in the last 3 months | 22.70 | 39.13 | 9.04 |  |
| 11 | Sleep in mattress vs beekeeper |  |  |  | 75.598*** |
|  | all family sleep in mat or polythene | 49.67 | 23.19 | 71.69 |  |
|  | only parents sleep in mattress, children on mat | 41.78 | 67.39 | 20.48 |  |
|  | sleep in bed and mattress even children | 8.55 | 9.42 | 7.83 |  |
| 12 | Marital status of household head vs beekeeper | | |  | 13.328*** |
|  | orphaned child youth | 2.96 | 0.72 | 4.82 |  |
|  | widow, single divorced | 6.91 | 2.17 | 10.84 |  |
|  | male or married woman | 90.13 | 97.10 | 84.34 |  |
| 13 | Age vs beekeeper |  |  |  | 0.219 |
|  | old | 26.32 | 26.81 | 25.90 |  |
|  | youth | 29.61 | 28.26 | 30.72 |  |
|  | middle-aged | 44.08 | 44.93 | 43.37 |  |
| 14 | Use of rare items like sugar, cooking oil vs beekeeper | | |  | 35.766*** |
|  | rarely buys sugar, meat or fries food | 39.80 | 23.19 | 53.61 |  |
|  | occasionally buys sugar, meat and fries food | 27.63 | 28.99 | 26.51 |  |
|  | frequently buys sugar, meat, fries food | 32.57 | 47.83 | 19.88 |  |
| 15 | Membership in any groups vs beekeeper | | |  | 21.562*** |
|  | unable to join even burial group | 6.91 | 13.04 | 1.81 |  |
|  | not in any farmer groups | 16.78 | 21.74 | 12.65 |  |
|  | in farmer group and other associations | 76.32 | 65.22 | 85.54 |  |
| 16 | Ownership of any scarce assets vs beekeeper | | |  | 4.913 |
|  | do not own any of the above | 22.70 | 28.26 | 18.07 |  |
|  | own small radio, bicycle | 44.08 | 42.75 | 45.18 |  |
|  | own mobile phone, motorcycle and bicycle | 33.22 | 28.99 | 36.75 |  |
| *** refers to significant at 1% level, and ** = significant at 5% | | | | | |
